# GenePy - a score for estimating gene pathogenicity in individuals using next-generation sequencing data

**DOI:** 10.1186/s12859-019-2877-3

**Published:** 2019-05-16

**Authors:** E. Mossotto, J. J. Ashton, L. O’Gorman, R. J. Pengelly, R. M. Beattie, B. D. MacArthur, S. Ennis

**Affiliations:** 1 Department of Human Genetics and Genomic Medicine, University of Southampton, Southampton, UK; 2 Institute for Life Sciences, University of Southampton, Southampton, UK; 3 Department of Paediatric Gastroenterology, Southampton Children’s Hospital, Southampton, UK

**Keywords:** Genome analysis, Mathematical modelling, Next-generation sequencing, Gene score, Pathogenicity score

## Abstract

**Background:**

Next-generation sequencing is revolutionising diagnosis and treatment of rare diseases, however its application to understanding common disease aetiology is limited. Rare disease applications binarily attribute genetic change(s) at a single locus to a specific phenotype. In common diseases, where multiple genetic variants within and across genes contribute to disease, binary modelling cannot capture the burden of pathogenicity harboured by an individual across a given gene/pathway.

We present GenePy, a novel gene-level scoring system for integration and analysis of next-generation sequencing data on a per-individual basis that transforms NGS data interpretation from variant-level to gene-level. This simple and flexible scoring system is intuitive and amenable to integration for machine learning, network and topological approaches, facilitating the investigation of complex phenotypes.

**Results:**

Whole-exome sequencing data from 508 individuals were used to generate GenePy scores. For each variant a score is calculated incorporating: i) population allele frequency estimates; ii) individual zygosity, determined through standard variant calling pipelines and; iii) any user defined deleteriousness metric to inform on functional impact. GenePy then combines scores generated for all variants observed into a single gene score for each individual.

We generated a matrix of ~ 14,000 GenePy scores for all individuals for each of sixteen popular deleteriousness metrics. All per-gene scores are corrected for gene length. The majority of genes generate GenePy scores < 0.01 although individuals harbouring multiple rare highly deleterious mutations can accumulate extremely high GenePy scores.

In the absence of a comparator metric, we examine GenePy performance in discriminating genes known to be associated with three common, complex diseases. A Mann-Whitney U test conducted on GenePy scores for this positive control gene in cases versus controls demonstrates markedly more significant results (*p* = 1.37 × 10^− 4^) compared to the most commonly applied association tool that combines common and rare variation (*p* = 0.003).

**Conclusions:**

*Per-gene per-individual* GenePy scores are intuitive when assessing genetic variation in individual patients or comparing scores between groups. GenePy outperforms the currently accepted best practice tools for combining common and rare variation. GenePy scores are suitable for downstream data integration with transcriptomic and proteomic data that also report at the gene level.

**Electronic supplementary material:**

The online version of this article (10.1186/s12859-019-2877-3) contains supplementary material, which is available to authorized users.

## Background

In the last decade, next-generation sequencing (NGS) has emerged as an effective tool for detecting single nucleotide variants (SNVs) causing rare conditions [1]. Recent retrospective studies have demonstrated an increase of 25–31% in diagnostic yield of rare diseases due to the application of exome or whole genome sequencing in a clinical framework [2, 3]. Through comparison against human genome reference sequence, high quality NGS data on individual patients can be used to identify variation in variant call files (VCF). These files typically contain in excess of 30,000 variants when based on whole exome data that captures sequence on the protein coding region of the genome only and runs to many millions when based on whole genome data. The successful identification of disease causing variation is critically dependent upon annotation and subsequent filtering of these data. Filtering strategies typically focus on very rare variants in panels of genes empirically implicated as related to the clinical manifestation or phenotype of interest. Further exclusion of synonymous variants that have no impact on protein amino acid sequence and variants that occur at a frequency substantially greater than that of the disease of interest are also deprioritised. These steps can reduce the search space for causal variation by orders of magnitude to smaller sets of hundreds or even tens of genetic changes that are then prioritised by in silico methods [4].

Many in silico tools have been developed in order to estimate the potential impact of genetic variants on gene/protein function. Predicting pathogenicity or deleterious impact can be achieved through a variety of algorithms that focus on one or more specific biological aspect(s). Three broad classes of deleteriousness prediction metrics are: (i) conservation metrics, (ii) function alteration metrics and (iii) composite scores. Conservation metrics such as GERP++ [5], phastCons [6] and phyloP [7] assign a high deleteriousness to variants where the homologous position in other species has remained constrained over evolutionary history. Scores focused on predicting the potential disruption of protein functionality, for example through alteration of resultant protein amino acid sequence, include SIFT [8], FATHMM [9], fathmm-MKL [10], PolyPhen2 [11], MutationTaster [12], PROVEAN [13] and VEST3 [14].

To date, no single in silico metric has proven unilateral superiority in estimating consequent severity, despite an expanding list [15] of metrics based on subtly different foundations and assumptions. While individual metrics have the ability to perform well in isolation, discordant evidence when assessing the same data with multiple metrics has led to increased uncertainty in choice of prediction tool [16]. This in turn has led to the development of a range of composite prediction tools applying statistical and machine learning methodologies that combine metrics assessing both conservation *and* functionality in order to obtain higher accuracy [17]. The most utilised composite scores include CADD [18], MetaSVM and MetaLR [19], M-CAP [20], Eigen [21], hyperSMURF [22] and DANN [23] with no one method emerging as optimal [24]. For this reason, when assessing variant deleteriousness it is still necessary to observe consensus prediction based on multiple scoring metrics rather than focusing on any single score [25]. This remains the case when studying rare Mendelian disease where single gene mutations imparting severe consequence are expected to represent the most extreme set of deleterious variants.

In contrast to rare diseases, common genetic diseases such as ischemic heart disease, asthma, inflammatory bowel disease (IBD) or Alzheimer’ disease are caused by the combined action of multiple genetic variants each differentially impacting risk and disease severity while working in combination with environmental exposures [26]. Collectively, common diseases impose an enormous economic burden and arguably have the greatest unmet need for diagnosis and stratified treatment [27]. The set of genes and variants imparting increased susceptibility vary from one patient to the next even when clinical presentation and molecular pathology appear indistinct.

Prior to transformative NGS approaches, genome-wide association studies (GWAS) made substantial advances in explaining the molecular bases of complex diseases. These studies tagged up to a million common single nucleotide markers across the genome and identified statistically significant distributions of bialleleic markers in large cohorts of independent patients compared to ethnically match controls. Genetic regions implicated by GWAS were assumed to harbour genes or regulatory elements underpinning the disease of interest. However, because these genetic breakthroughs were achieved using necessarily huge cohorts of patients compared to controls, while their findings hold true for massive patient groups, they are largely uninformative on an individual patient basis. Importantly, the relevance and value of GWAS findings to individual patients has therefore not translated through to clinical practice in terms of either diagnosis or treatment.

Application of NGS to improve our understanding of common oligogenic diseases have been largely limited to burden tests that extend the association testing framework to integrate information about common and rare variation across discrete genomic regions such as genes. While this approach harnesses the power of NGS through inclusion of rare variants that can only be detected by sequencing approaches, they are most often implemented through collapsing multiple variants into a single value for univariate analysis. The limited success of these approaches are partly attributed to their intrinsic lack of biological information and inclusion of both causal and benign genetic variation [28, 29]. In order to overcome this limitation, Neale et al. developed the C-alpha test, correcting for both protective and deleterious variants but at the cost of losing statistical power. Currently, SKAT (and SKAT-O optimised for small sample size) [30] represents the most sensitive approach to test for association between a genomic region and a phenotype. SKAT jointly assesses both rare and common variants maximising the statistical power and representing a new class of analysis lying between burden and association tests and has been successfully applied to a large variety of complex diseases [31–35].

While NGS is proving a transformative technology for the diagnosis and treatment of rare diseases, its relatively modest application in common diseases is limited by a lack of analytical approaches that incorporate *individual* profiles of genetic variation ascertained through NGS annotated with biologically meaningful information on their frequency and consequence.

Instead of variant focussed approaches typical for rare disease or large cohort approaches that distinguish GWAS, contemporary analyses of complex polygenic disorders require the development of tools that combine both mutational burden and biological impact of a personalised set of mutations into single scores for discrete sub-genomic units such as genes. A matrix of such a set of scores for any one individual could then be analysed using various methodology including machine learning.

In this study, we describe the development and implementation of GenePy, a novel gene-level scoring system for integration and analysis of next-generation sequencing data on a per-individual basis. The goal of the GenePy scoring system is not to create a statistical tool for burden or association tests, but to generate a novel scoring system that transforms NGS data interpretation from variant level to gene level. The aim is to enable a gene based scoring system for individuals that can be used to compare single gene pathogenicity between individuals or to prioritise genes with high pathogenic loading for scrutiny for any single individual. In addition, GenePy aims to increase the intrinsic biological information content by incorporating data on allele frequency and observed zygosity in addition to any user-defined variant deleteriousness metric. The GenePy scoring system aims to transform typical sequencing data output into a format suitable for integration into downstream network analyses or machine learning approaches for stratification. In the absence of other comparator scoring systems, we validate GenePy performance on three complex diseases: paediatric inflammatory bowel disease (IBD), Parkinson’s disease (PD) and primary open angle glaucoma (POAG).

## Implementation

### Sample data

Whole exome sequencing (WES) data were derived from two sources. This first group comprised 309 patients diagnosed in childhood with IBD. This cohort (further described in [36]) includes unrelated, Caucasian patients ascertained and recruited through Southampton Children’s Hospital who were diagnosed under the age of 18 years according to the modified Porto criteria [37]. Additional WES data from a cohort of 199 anonymised individuals diagnosed with an infectious disease but unselected for any form of autoimmune disease were also used to give a total cohort size of 508 individuals with WES data.

Genomic DNA was extracted from peripheral venous blood and fragmented DNA subjected to adaptor ligation and exome library enrichment using the Agilent SureSelect All Exon capture kit versions 4, 5 and 6. Enriched libraries were sequenced on Illumina HiSeq systems.

### WES data processing

Raw sequencing fastq sequencing data from all 508 samples were processed using the same custom pipeline. VerifyBamID [38] was utilised to check the presence of DNA contamination across our cohort of 508 individuals. Alignment was performed against the human reference genome (GRCh38/hg38 Dec. 2013 assembly) using BWA [39] (version 0.7.12). Aligned BAM files were sorted and duplicate reads were marked using Picard Tools (version 1.97). Following GATK v3.7 [40] best practice recommendations [41], base qualities were recalibrated in order to correct for systematic errors produced during sequencing. Finally, variants were called using GATK HaplotypeCaller was applied to produce a gVCF file for each sample. Samples were processed on the University of Southampton IRIDIS cluster requiring an average of 4 h run time per sample on a 16-processor node.

While the standard VCF format reports only alternative calls, the gVCF format identifies non-variant blocks of sequencing data and returns reference calls for loci therein. This enables affirmative calling of homozygous reference loci when combining call sets from multiple samples. Multi-sample variant calling was achieved through calling each individual sample separately and then merging all gVCFs using GATK GenotypeGVCFs. Processing efficiency was optimised for the set of 508 individual samples through batching into six subsets using GATK’s CombineGVCFs (approx. 6 h/batch on a 16 processor node) and the resultant six gVCF files were merged for genotyping with GenotypeGVCFs (approx 1 h on a 16 proc. node). Annotation of this composite file applied Annovar v2016Feb01 using default databases refSeq gene transcripts (refGene), deleteriousness scores databases (dbnsfp33a) and dbSNP147). Variant allele frequencies were sourced through Annovar (ExAc03 [42]) or ensembl human variation API [43] where ExAc data were missing.

### Quality control framework

In order to reduce heterogeneity, it is necessary to control for bias encountered due to alternative capture kit versions and variant quality. For the entire cohort of 508 samples, exon enrichment was performed using Agilent SureSelect capture kits but at different time-points. For this reason, there is inter-capture kit variability across the 508 cohort with kit versions 4, 5 and 6 being applied. To correct for disparity in the regions targeted by respective versions, all downstream analyses were restricted to the set of overlapping targeted genomic locations (as defined by respective kit BED files) using BEDtools v2.17 [44].

Following GATK best practice guidelines, HaplotypeCaller default settings were utilised, implying that only variants with a minimum Phred base quality score of 20 were called.

### GenePy score

Individuals typically have multiple variants across the coding region of genes making the interpretation of their combined effect challenging. We hypothesised that for each individual sample *h* within our cohort *H = {h*_*1*_*, h*_*2*_*, …, h*_*n*_*},* the loss of integrity of any given gene *g* in the RefGene database *G* = {g_1_, g_2_, … g_m_} can be quantified as the sum of the effect of all (*k*) variants within its coding region observed in that sample, where each biallelic mutated locus (*i*) in a gene is weighted according to its predicted allele deleteriousness (*D*_*i*_), zygosity and allelic frequency (*f*_*i*_). The GenePy score *S*_*gh*_ for a given gene (*g*) in individual (*h*) is

$$ {S}_{gh}=-\sum \limits_{i=1}^k{D}_i{\log}_{10}\left({f}_{i1}\bullet {f}_{i2}\right) $$

At any one variant locus (*i*), we represent both parental alleles using *f*_*i*1_ and *f*_*i*2_ to embed the population frequency of allele_1_ and allele_2_ and, in doing so, model observed biological information on both frequency and zygosity. Any homozygous genotype therefore is simply the observed allele frequency squared whereas the product of each of the observed alleles is calculated for heterozygous genotypes. The latter can therefore accommodate variant sites with multiple alleles in addition to the typically encountered bialleleic single nucleotide polymorphisms (SNPs). Hemizygotic variation from male X-chromosomes are treated as homozygotic. Where a variant may be novel to an individual or absent from reference databases, we impose a lower frequency limit of 0.00001. This lower limit is arbitrarily set to conservatively reflect the lowest frequency that can be observed in the largest current repository of human variation (ExAc03). The log function is applied to upweight the biological importance of rare variation.

The GenePy algorithm represents a genetic mixed model, combining the known multiplicative effect of two alleles at a single diploid locus [45] (the frequencies of both observed alleles are multiplied) but with an additive effect at the gene level (variant scores are summed within a gene). The contribution of all variation within a gene is modelled in this additive fashion in order to enable the cumulative pathogenicity incurred from the effects of multiple small/modest effects imposed by individual mutations thus reflecting the non Mendelian inheritance pattern in common diseases. An additive model is assumed to be most universally applicable model particularly in the non-Mendelian situation relevant to many common diseases [46].

Deleteriousness metrics were developed to assess damage induced by nonsynonymous variation, therefore structural variants such as frameshifts or stop mutations that truncate proteins are not routinely assigned deleteriousness values. Due to their highly detrimental impact to function we assign all protein truncating mutations the maximal deleteriousness value of 1. Synonymous and splicing variants are not routinely annotated by ANNOVAR and were not included in the current assessment.

Importantly, the choice of variant deleteriousness score is user-defined, and therefore the GenePy score is able to take into account different definitions of pathogenicity depending on context. Herein we examine the relative attributes of using any one of sixteen of the most commonly applied scores (Table 1). Sixteen of the most common deleteriousness (D) metrics were selected for implementation within the GenePy algorithm. Five of these metrics (shown in bold) are unbounded. In order to implement unbounded metrics in GenePy it was necessary to impose lower and upper limits by applying the respective minimum and maximum values observed in the dbnsfp33a database of 83,422,341 known SNV mutations. These limits were used to transform observed values in our cohort scaled to 0–1.

**Table 1:** Pathogenicity scores for SNVs and their reported ranges in the dbsnfp database

Metric	Type	Implementation	Actual range	Imposed range for transformation
CADD	Composite	Score	**-∞** to **+∞**	−7.53 to 35.79
DANN	Composite	Score	0 to 1	–
FATHMM^a^	Functionality	1-Score	**-∞** to **+∞**	−16.13 to 10.64
fathmm-MKL	Composite	Score	0 to 1	–
GERP++_RS	Conservation	Score	**-∞** to **+∞**	−12.3 to 6.17
M-CAP	Composite	Score	0 to 1	–
MetaLR	Composite	Score	0 to 1	–
MetaSVM	Composite	Score	**-∞** to **+∞**	−2 to 3
MutationTaster^a^	Functionality	1-Score if N/P; Score if A/D	0 to 1	–
phastCons	Conservation	Score	0 to 1	–
phyloP	Conservation	Score	**-∞** to **+∞**	−13.28 to 1.2
Polyphen2_HDIV	Functionality	Score	0 to 1	–
Polyphen2_HVAR	Functionality	Score	0 to 1	–
PROVEAN^a^	Functionality	1-Score	−14 to 14	–
SIFT^a^	Functionality	1-Score	0 to 1	–
VEST3	Functionality	Score	0 to 1	–

^*a*^In order to maintain uniform directionality, the complement (1 – score) of a value was taken so that across scores, a value of 0 consistently indicated benign variation and a value of 1 inferred maximal pathogenicity

As a function of their size alone, larger genes have greater opportunity to accrue higher deleterious GenePy scores through having a greater number of variants thus inflating GenePy scores. We therefore generated GenePy scores corrected for the length of targeted gene regions (GenePy_cgl_) by dividing the GenePy score by the targeted length in base pairs and then multiplying by the median observed targeted gene length in our data (1461 base pairs). A final set of 16 deleteriousness metrics, each with a range of 0–1 where highest values were most deleterious, were individually implemented in the model.

### GenePy score validation on the IBD dataset

In the absence of any comparable gene based scoring system for individuals, GenePy performance was benchmarked by assessing the power to determine significantly different score distributions in disease cases compared to controls for a known causal gene through a Mann-Whitney U test. Using the same variant data, the statistical difference in GenePy scores was compared against that of SKAT-O - the most commonly applied gene level association test. The cohort comprised 309 individuals diagnosed with inflammatory bowel disease (IBD) and 199 controls unselected for autoimmune conditions. The analysis focussed on the *NOD2* gene - the most strongly and repeatedly associated common disease gene conferring strong association specifically with the Crohn’s disease (CD) subtype of IBD [47–49]. *NOD2* was selected as a positive control gene, whereby evidence for increased burden of deleterious mutation encoded in CD patient DNA compared to either ulcerative colitis (UC) or control DNA is expected.

The matrix of *NOD2* GenePy scores calculated for all 508 samples was split into controls and cases with the latter further divided into UC and CD subtypes. Statistical significance of GenePy score distribution difference between groups was calculated using the Mann-Whitney U test for unpaired data. Using the same variant input data, the SKAT-O gene based test for association was performed twice using default settings: firstly by considering all variants called within *NOD2* and secondly including only rare variants (MAF < 0.05) as per developer recommendations [30].

Association tests succumb to false positive results due to spurious association brought about by population stratification or systematic differences in case versus control data. We excluded non-Caucasian individuals identified through comparison against the 1000 Genomes Project [50] using Peddy software [51] for ethnic imputation. We enforced parity in sequencing depth (known to impact power to call genetic variation [52]) for case-control data by limiting all score validation data to variants called in gene regions with a minimum read depth of 50X.

### GenePy score validation on the Parkinson’s disease dataset

A second validation of the GenePy score was performed using WES from the Parkinson’s Progression Marker Initiative (PPMI) [53]. Six hundred and ten Caucasian patients diagnosed with Parkinson’s disease (PD) were selected from this cohort. No control data were generated within this cohort.

Parkinson’s disease is a common complex condition involving the central nervous system. Disease aetiology is complex and only partially understood, but the increased risk of occurrence driven by family history of disease indicates a strong genetic component [54]. To date, several genes have been associated with Parkinson’s disease, however only few have been validated as disease causing. In our approach, we focussed on the panel of six genes routinely tested in clinical settings: *LRRK2*, *PRKN* (*PARK2*), *PARK7*, *PINK1*, *SNCA* and *VPS35*. The gene panel and technical notes are further described the UK Genetic Testing Network database (https://ukgtn.nhs.uk).

Whole exome sequencing data for this cohort was generated using Illumina 2500 sequencing machines and Nextera Rapid Capture Expanded Exome Kit. Raw sequencing data were processed as per those for the IBD cohort. GenePy scores, implementing the CADD deleteriousness metric (given CADD’s high performance and more complete gene annotation), were generated for 610 PD samples for the six genes included in the panel. GenePy distributions in PD cases were compared using a Mann-Whitney U test against non-PD samples. In the absence of within-cohort control data, IBD and control samples described above were used as non-PD controls for these tests. In order to assure compatibility, GenePy scores were calculated only for common regions targeted by both Nextera and Agilent exon enrichment capture kits used by the respective studies (intersection of bed files). Statistical significance was compared with results obtained through a SKAT-O test as previously described.

We further tested the ability of GenePy to detect extreme gene differences between PD patients and non-PD individuals. A one-tailed Mann-Whitney U test was conducted between the highest 5% of the GenePy distribution scores from the PD patients and the highest 5% of the non-PD cohort for each gene investigated.

### GenePy score validation on the primary open angle Glaucoma cohort

The third validation of GenePy was performed on a cohort of Caucasian patients (*n* = 358) affected by primary open angle glaucoma (POAG) [55], a glaucoma subtype characterised by an open and normal anterior chamber angle, increased intraocular pressure and no other concurrent adverse phenotypes [56]. POAG is a common complex condition with a strong genetic component with first-degree relatives of affected individuals harbouring an eightfold increased risk [57]. Previous studies have established *MYOC* as causative gene in approximately 3% of the POAG diagnoses [58].

Sequencing data for the POAG cohort were generated using Nextera Rapid Capture Custom Enrichment kit, the Nextera 500 sequencing platform and the same best practice bioinformatic pipeline as applied in the IBD cohort [59].

Mann-Whitney U was applied to test whether GenePy was capable of detecting a statistically significant difference between the POAG cohort and non-POAG samples (using IBD and control samples as a proxy for matched controls as above) within the *MYOC* gene. Regions common to the Nextera Rapid Capture Custom Enrichment kit and Agilent SureSelect Capture chemistries were selected using bed file data to ensure compatibility of GenePy scores.

The difference between extreme GenePy scores in the POAG patients compared to non-POAG individuals was assessed. Given the known frequency of *MYOC* pathogenic mutations of 3%, statistically significant differences within the extreme top 3% distribution of both groups was compared as above.

## Results

### QC results

All WES data (*n* = 508, n_ibd_ = 309, n_ctrl_ = 199) underwent quality control assessment for contamination using VerifyBamID and were confirmed free of contamination (free-mix statistic < 0.01). Out of 508 individuals, we identified three pairs of first degree relatives, one set of monozygotic twins and one mother-father-child trio. In order to correct for relatedness, which would bias association tests, for each pair, the sample with poorest coverage data was excluded. For the trio, the child data were excluded and unrelated parents retained.

### GenePy score behaviour – impact of allele frequency and zygosity

**Fig. 1:**
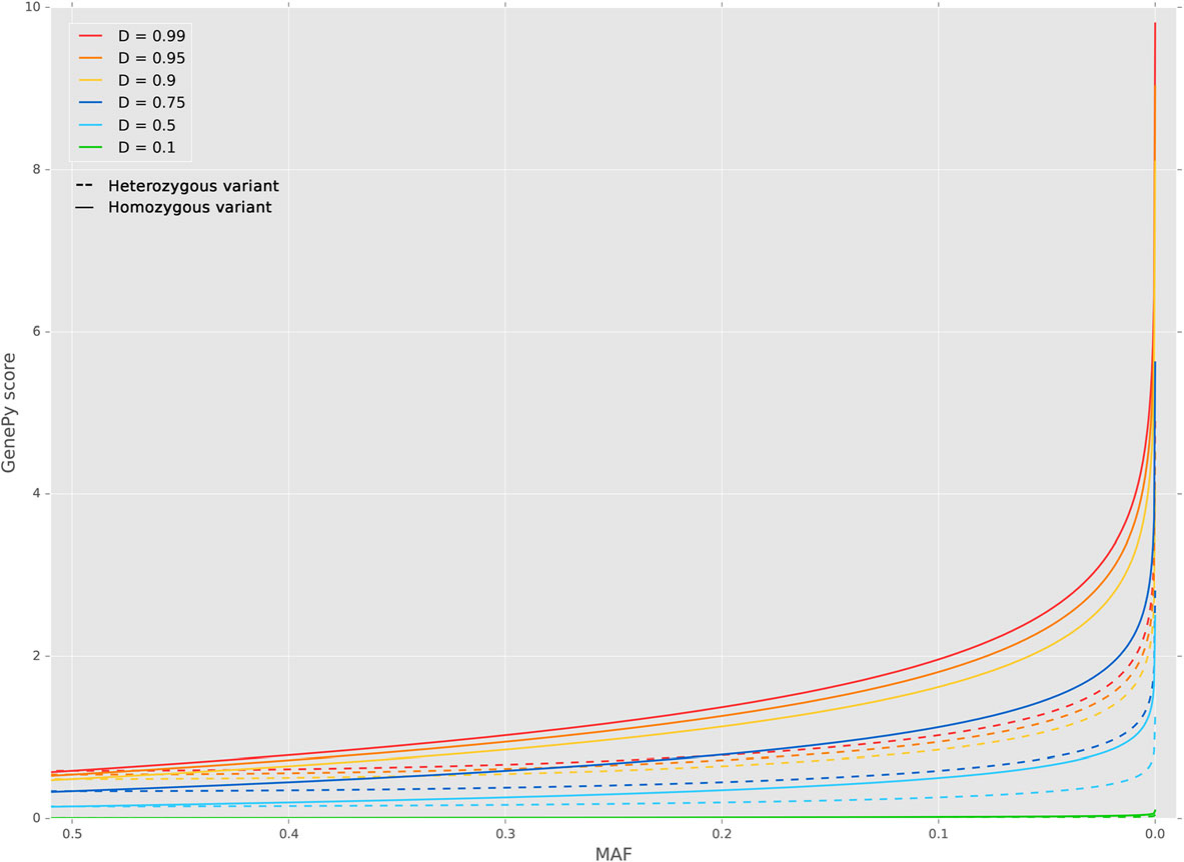
Single variant GenePy score distribution under fixed deleteriousness values. Impact of varying zygosity and minor allele frequency (MAF)

Figure 1 shows the results of simulated GenePy score (y-axis) calculated across a range of deleterious metric scores (0.1, 0.5, 0.75, 0.9, 0.95, 0.99) with varying minor allele frequency (x-axis) and further depicts the consequence of heterozygote versus homozygote states. The plot reveals the logarithmic nature of GenePy scores for a single locus only (whereas for any individual, their per gene GenePy score is weighted sum of all variant scores observed in that individual across that gene). For any single variant, the theoretical maximum observable GenePy value of ten occurs only with highest deleteriousness value (*D*), the lowest minor allele frequency (MAF = 0.00001) and in the homozygous state whereas the upper limit for a heterozygote with the same deleteriousness and frequency settings is five. The logarithmic scale implemented in GenePy algorithm confers rapidly increasing scores as the MAF approaches novelty.

### GenePy score behaviour – impact of deleteriousness metric

While there are 27,238 genes annotated in RefSeq, we aimed to generate GenePy scores only for the overlapping subset of 21,577 target genes captured by all versions of the SureSelect capture kits applied. The GenePy scoring algorithm was executed for each of sixteen commonly applied metrics (Table 1). There is fluctuation in the number of genes for which variants were annotated with deleteriousness metric data using ANNOVAR ranging from 12,921 for M-CAP (one of the most recently released scores) to 14,745 genes annotated scores for Polyphen2_HDIV (one of the earliest developed deleteriousness scores) (Table 2). Among the 508 individuals that underwent GenePy scoring of exome data, the majority of genes are invariant within any one individual (e.g. median 9917 for CADD metric). This is expected for intrinsically sparse genomic data. However, across the cohort, no single gene returns a GenePy score of zero in all individuals indicating all genes have at least one rare variant observed amongst the 508 individuals. The vast majority of genes are scored with GenePy values of less than 0.01 and correction for gene length marginally increases the number of genes achieving lowest scores. More than 97% of genes achieve a score of less than 0.01 when the M-CAP metric is used whereas FATHMM scores approximately 65% of genes in the 0–0.01 range. The inflated percentage of invariant genes observed when implementing M-CAP is explained by its tendency to depress weight for benign variants compared to other tested metrics [20].

**Table 2:** Statistical attributes of whole gene GenePy scores computed for sixteen deleteriousness metrics. Number of genes for which GenePy scores were calculated, median number of non-variant genes (GenePy = 0), mean GenePy scores, mean and standard deviation across our cohort (*n* = 508), coefficient of variation (CV, defined as *σ*/*μ*) and the median number of genes with a GenePy score < 0,01 as percentage of the total number of genes. The same information is reported for GenePy_cgl_

Metric	Gene scores calculated	^a^Median no. of genes with GenePy = 0 within individuals (%)	Max GenePy	MeanGenePy	CV _uncorrected_	Median no. of genes with GenePy < 0.01 within individuals (%)	Max GenePy_cgl_	Mean GenePy_cgl_	CV_cgl corrected_	Median no. of genes with Genepy_cgl_ < 0.01(%)
CADD	14,184	9917 (69.92%)	32.15	0.10	3.81	10,231 (72.13%)	74.19	0.08	8.09	10,304 (72.64%)
DANN	14,184	9917 (69.92%)	110.48	0.33	3.37	10,153 (71.58%)	304.15	0.25	6.96	10,196 (71.88%)
FATHMM	13,143	9981 (75.94%)	72.73	0.16	4.15	10,923 (83.11%)	269.62	0.11	6.42	11,092 (84.40%)
fathmm-MKL	14,178	9039 (63.75%)	50.10	0.16	3.29	9282 (65.48%)	131.34	0.12	7.55	9332 (65.84%)
GERP++_RS	14,197	9910 (69.80%)	100.44	0.32	3.35	10,116 (71.25%)	283.69	0.24	6.47	10,143 (71.44%)
M-CAP	12,921	12,577 (97.34%)	24.52	0.02	12.65	12,596 (97.48%)	59.88	0.02	19.05	12,630 (97.74%)
MetaLR	14,063	12,752 (90.68%)	38.14	0.04	8.77	13,146 (93.48%)	87.80	0.04	16.14	13,253 (94.24%)
MetaSVM	14,076	9845 (69.94%)	36.76	0.10	3.95	10,141 (72.04%)	99.44	0.08	8.94	10,207 (72.51%)
MutationTaster	14,039	12,161 (86.62%)	90.86	0.13	5.24	12,521 (89.19%)	332.05	0.09	9.02	12,579 (89.60%)
phastCons	14,197	10,217 (71.97%)	100.64	0.21	3.79	11,018 (77.60%)	324.41	0.14	5.76	11,116 (78.29%)
phyloP	14,202	9910 (69.78%)	118.81	0.40	3.31	10,107 (71.17%)	332.05	0.31	7.15	10,131 (71.34%)
Polyphen2_HDIV	14,745	11,824 (80.19%)	65.48	0.14	4.89	12,558 (85.16%)	257.00	0.12	12.08	12,658 (85.84%)
Polyphen2_HVAR	14,741	11,470 (77.81%)	59.67	0.11	5.47	12,621 (85.62%)	239.71	0.09	14.03	12,778 (86.69%)
PROVEAN	13,888	9733 (70.08%)	74.16	0.23	3.37	9958 (71.70%)	219.39	0.17	7.93	10,003 (72.02%)
SIFT	14,561	11,088 (76.15%)	99.69	0.25	3.69	11,224 (77.08%)	265.64	0.20	7.04	11,257 (77.31%)
VEST3	14,170	9919 (70.00%)	53.36	0.09	5.69	10,528 (74.29%)	136.56	0.08	12.56	10,821 (76.36%)

^a^Across the cohort of 508 individuals assessed, individual samples have a very high median number of invariant genes resulting on GenePy scores of zero

Across the ~ 14,000 genes achieving GenePy scores, the observed score mean (uncorrected for length) in our cohort of 508 samples ranges from 0.02 to 0.40 depending on the applied deleteriousness metric. Correction of all scores for gene length has only a modest effect on the range of the mean scores observed (0.02–0.31), however, gene length correction increases the spread of the data reflected by an approximate two-fold increase in the coefficient of variation (CV) for GenePy scores observed across all sixteen deleteriousness metrics. This is despite the fact that for all deleteriousness metrics, correction for gene length subtly increases the proportion of genes with lowest scores confirming that genes of exceptional size incurred inflated scores due to length. GenePy scores generated with M-CAP are least impacted by gene length correction but maintain the largest CV.

**Fig. 2:**
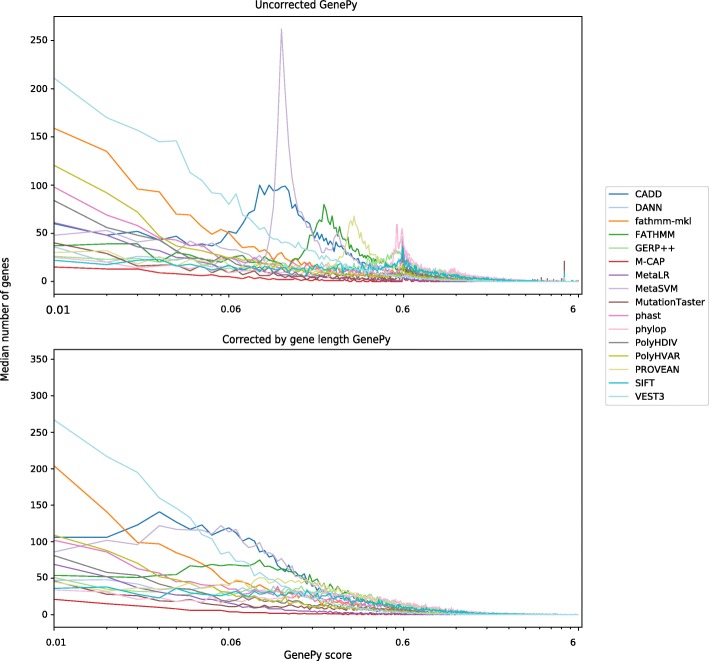
GenePy profiles observed for all genes across the whole cohort for all sixteen deleteriousness metrics. Uncorrected GenePy scores (upper panel) exhibit characteristic spikes reflecting gene scores strongly influenced by the effect of: single highly deleterious (D = 1) common homozygous variants (red) or; single highly deleterious very rare/novel variants (MAF = 0.00001) (blue). GenePy_cgl_ score profiles (lower panel) do not display these spikes. Invariant genes conferring a GenePy score < 0.01 are overrepresented and not shown here by commencing the x-axis with the 0.01–0.02 bin. All sixteen versions of the GenePy score exhibit long tails in the GenePy score distribution truncated here at a score of six

In order to further investigate the behaviour of GenePy scores across genes, we calculated the median number of genes exhibiting scores falling within non-overlapping bins across the entire cohort. Figure 2 shows the profiles for the 0.01 to 6 range of GenePy scores and a bin size of 0.01. Genes with scores < 0.01 are overrepresented (Table 2) and not shown. Across most of the sixteen metrics, a distinct pattern characterised by two spikes around uncorrected GenePy scores of 0.6 and 5 represent genes strongly influenced by a single highly deleterious common homozygous variants (*D = 1*, MAF = 0.5) or a single highly deleterious very rare heterozygous variant (*D = 1*, MAF = 0.00001) respectively. This profile was apparent for most deleteriousness metrics (except CADD, FATHMM, MetaSVM and VEST3, see Additional file 1: Figure S1). These two distinctive spikes are not observable once GenePy scores are corrected for the targeted gene length (Fig. 1, lower panel and Additional file 1: Figure S2). We did not observe further spikes or other anomalies in the long right tail of the distribution of scores greater than 6.

**Fig. 3:**
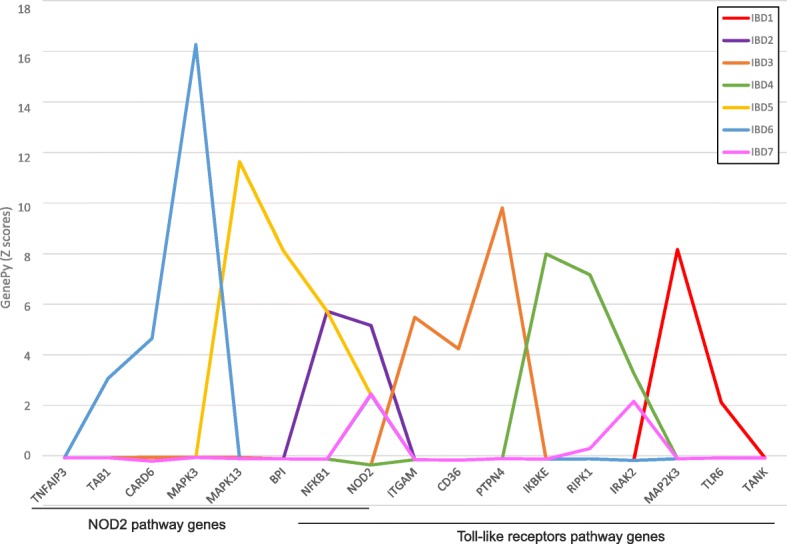
GenePy score profiles for seven independent patients diagnosed with IBD across selected genes from the NOD2 and TLR pathways. GenePy scores shown were implemented using the M-CAP deleteriousness (D) metric. To facilitate plotting, raw GenePy scores were transformed to Z-scores for each gene. Different colours depict individual patient profiles. Despite being diagnosed with the same disease, all individuals exhibit distinctive profiles across key genes implicated in key immune pathways. Some individuals have evidence of gene pathogenicity within the same pathway (e.g. IBD5 and IBD6) this is conferred through accumulated mutation in different genes – IBD6 has elevated gene-level scores for TAB1, CARD6 and MAPK3 while IBD5 may have impaired function in this pathway due to combined mutation in MAPK13, BP1 and NFKB1. Similarly, IBD1, IBD3 and IBD4 exhibit pathogenic profiles in TLR pathway genes only. These individual level data can be combined with disease phenotype, severity and treatment outcome data in machine learning models to better stratify patient cohorts and realise the promise of personalised medicine

For a subset of 6 patients we plot the gene-level scores for 17 genes across two different molecular pathways important to immune function (Fig. 3). This graphically demonstrates how individual patients diagnosed with the same non-Mendelian condition have unique gene-level deleteriousness score profiles. Individual patients can be genetically compromised within the same or distinct molecular pathways.

### GenePy score validation - IBD cohort

Bias conferred by *NOD2* gene coverage, related samples and non-Caucasian ethnicity (Additional file 1: Figure S3) was removed from all IBD cases (*n* = 6_<50x_, *n* = 1_relative_ and *n* = 20_non-Caucasian_) and non-IBD control samples (*n* = 16_<50x_, *n* = 4_relatives_ and *n* = 13_non-Caucasian_) respectively. There remained 282 IBD cases for analysis of which 172 were diagnosed with Crohn’s disease, 100 with ulcerative colitis and a further 10 patients had a diagnosis of IBD undetermined (IBDU). There was a corresponding number of 166 controls.

The *NOD2* GenePy scores for the 282 IBD and 166 control individuals were calculated using all sixteen deleteriousness metrics. (Additional file 1: Figure S4). Given *NOD2* gene variant association is specific to the CD subtype of IBD, we calculated GenePy scores for both subtypes and grouped separately (Additional file 1: Table S1).

The Mann-Whitney U test comparison of the distribution of *NOD2* GenePy scores between all IBD, CD and UC subtypes against controls identified statistically significant differences (Table 3). Only modestly significant differences for just three of the implemented deleteriousness metrics (M-CAP, fathmm-mkl and MutTaster) were observed comparing all IBD against controls in this relatively small sample. When the cases were stratified by disease subtype, UC samples had significantly lower GenePy scores compared to controls but only for two of the implemented deleteriousness metrics (MetaLR, phastCons). As expected, the most significant difference in *NOD2* score distribution was observed when comparing CD patients only against controls. Without exception, a highly significant difference was observed using every deleteriousness metric with M-CAP the most significant (*p* = 1.37 × 10^− 4^) all of which would withstand correction for the three independent tests performed. Regardless of which deleteriousness metric is used, the mean GenePy score is consistently higher in CD patient when compared with controls.

**Table 3:** NOD2 GenePy score statistics (maxima and means) and Mann-Whitney U tests across groups for all sixteen deleteriousness metrics. *p*-values smaller than 1 × 10^−2^ or smaller than 5 × 10^−2^ are highlighted by two (**) or one (*) asterisks respectively. SKAT-O gene association results comparing patient groups against controls provided below thick line

Metric	Controls (*n* = 166)		IBD (*n* = 282)			UC (*n* = 100)			CD (*n* = 172)		
	*max*	*mean*	*max*	*mean*	*Mann-Whitney U comparison against controls*	*max*	*mean*	*Mann-Whitney U comparison against controls*	*max*	*mean*	*Mann-Whitney U comparison against controls*
CADD	2.71	0.28	3.52	0.40	1.04 × 10^−1^	2.66	0.20	1.38 × 10^−1^	3.52	0.54	4.62 × 10^−4^ **
DANN	5.92	0.84	7.62	1.06	1.36 × 10^−1^	5.62	0.57	1.22 × 10^−1^	7.62	1.38	8.16 × 10^− 4^ **
FATHMM	3.33	0.49	4.34	0.66	1.04 × 10^−1^	3.14	0.38	1.47 × 10^− 1^	4.34	0.84	4.84 × 10^− 4^ **
fathmm-MKL	4.53	0.37	6.24	0.55	4.54 × 10^−2^ *	3.78	0.25	3.15 × 10^− 1^	6.24	0.76	1.79 × 10^− 4^ **
GERP++_RS	5.30	0.64	7.00	0.87	1.26 × 10^− 1^	4.95	0.42	1.27 × 10^− 1^	7.00	1.17	6.95 × 10^− 4^ **
M-CAP	1.87	0.12	3.39	0.22	1.58 × 10^− 2^ *	1.73	0.08	4.62 × 10^− 1^	3.39	0.32	1.37 × 10^− 4^ **
MetaLR	2.42	0.16	3.39	0.29	2.71 × 10^− 1^	1.81	0.10	2.34 × 10^− 2^ *	3.39	0.42	1.63 × 10^−3^ **
MetaSVM	2.67	0.30	3.61	0.43	9.88 × 10^− 2^	2.50	0.22	1.50 × 10^− 1^	3.61	0.57	4.39 × 10^− 4^ **
MutationTaster	4.38	0.26	5.10	0.39	4.48 × 10^− 2^ *	2.65	0.13	4.37 × 10^− 1^	5.10	0.57	7.47 × 10^− 4^ **
phastCons	4.66	0.35	5.24	0.56	2.86 × 10^− 1^	3.54	0.24	2.70 × 10^− 2^ *	5.24	0.77	2.16 × 10^− 3^ **
phyloP	6.32	1.02	7.93	1.27	1.23 × 10^− 1^	5.92	0.75	1.38 × 10^− 1^	7.93	1.62	7.09 × 10^− 4^ **
Polyphen2_HDIV	5.32	0.68	7.03	0.82	2.02 × 10^− 1^	2.30	0.33	6.22 × 10^− 2^	7.03	1.13	1.20 × 10^− 3^ **
Polyphen2_HVAR	4.86	0.46	5.31	0.64	1.65 × 10^− 1^	2.07	0.21	7.22 × 10^− 2^	5.31	0.92	7.90 × 10^− 4^ **
PROVEAN	4.33	0.66	5.23	0.86	1.04 × 10^− 1^	4.08	0.49	1.45 × 10^− 1^	5.23	1.10	4.84 × 10^− 4^ **
SIFT	5.91	0.95	7.61	1.14	1.47 × 10^− 1^	5.43	0.64	1.16 × 10^− 1^	7.61	1.47	9.64 × 10^− 4^ **
VEST3	3.28	0.30	4.21	0.44	1.36 × 10^− 1^	2.24	0.17	1.13 × 10^− 1^	4.21	0.62	7.48 × 10^− 4^ **
SKAT-O (all variants)	–	–	5.41 × 10^− 1^			9.76 × 10^− 2^			3.46 × 10^− 2^ *		
SKAT-O (MAF < 0.05)	–	–	4.63 × 10^− 1^			1.37 × 10^− 1^			5.02 × 10^− 2^		

Interestingly, similar results were observed for the SKAT-O gene test of association when using all variant frequency data but lost significance when restricted to rare variation (MAF < 0.05). Importantly, the magnitude of the difference between CD patients and control groups was statistically weaker (*p* = 0.0346) and less robust to correction for multiple testing.

Although not the purpose of this comparison, we confirmed GenePy whole gene comparison provided statistical evidence two orders of magnitude greater than any single variant association result (Additional file 1: Table S1).

### GenePy score validation - Parkinson’s disease cohort

Of the six genes investigated for different GenePy distributions between the PD cohort (*n* = 610) and the non-PD (*n* = 465) cohort, statistically significant results were observed for the *PINK1* gene only (*p* = 0.013) (Table 4). The SKAT-O test did not detect significant associations for any of the six genes.

**Table 4:** Comparison of PD versus non-PD individuals. Significant results are shown in bold type. For each gene the most significant result only of all SNV association tests is shown and for each these the rs id is provided. Additionally, the number of SNV association test conducted within each gene is indicated in brackets. No correction is made for testing of six genes nor for testing multiple SNVs within any given gene

Test PD vs non-affected samples	*LRRK2*	*PARK7*	*PINK1*	*PRKN*	*SNCA*	*VPS35*
GenePy	0.178	0.445	**0.013**	0.983	0.828	0.206
SKAT-O	1	0.557	0.157	0.427	0.712	0.741
Top 5% comparison	**0.002**	0.107	**0.010**	**0.021**	0.347	**0.036**
Most significant SNV (# tested)	**0.034** rs10878245 (88)	0.081 rs71653621 (6)	**0.042** rs148871409 (21)	0.051 rs1801582 (27)	0.433 rs548523899 (7)	0.433 rs168745 (17)

Restricting the analysis to just the extreme right tail of the GenePy distribution for each of the six PD genes, statistically significant differences were observed between PD and non-PD individuals for *LRRK2* (*p* = 0.002), *PINK1* (*p* = 0.010)*, PRKN* (*p* = 0.021) and *VPS35* (*p* = 0.036). Patients with severe *PINK1* and *PRKN* mutations present early onset forms of Parkinson’s disease and have been reported in this PD cohort [60]. The most significant result for each gene from traditional single variant association tests reported significant results for two genes only -*LRRK2* (rs10878245, *p* = 0.034) and *PINK1* (rs148871409, *p* = 0.042) although this required the analysis of multiple SNVs (see Table 4) within each gene.

### GenePy score validation - primary open angle glaucoma (POAG) cohort

Comparison of GenePy scores between the POAG cohort (*n* = 358) and the non-POAG cohort (*n* = 465) did not reveal a statistically significant difference for the *MYOC* gene (*p* = 0.18). Similarly, significance was not detected using SKAT-O methodology (*p* = 0.66).

However, performing a Mann-Whitney U test of GenePy scores between the extreme end of the right tail of the GenePy distribution (this time limited to 3% to reflect the known biology) of the POAG cohort and the top 3% of the non-POAG cohort, we observed a statistically significant difference (*p* = 0.048).

In a single variant association test framework, 18 SNVs within the *MYOC* gene were tested for association and only one (rs61730974) reached statistical significance without correcting for multiple testing (*p* = 0.0318).

## Discussion

Next generation sequencing is a disruptive technology set to transform biological assessment. Globally, it is rapidly integrating into the medical sector with numerous countries already funding whole genome sequencing of patient samples for diagnosis and treatment of rare disease and cancer. Multiple metrics have emerged that aim to annotate individual mutations with a view to sensitively implicating causal versus non-causal variation. However, for common complex diseases where the action of an unknown number of multiple variants converge to increase susceptibility, the molecular assessment of mutation profiles is necessarily less binary. Furthermore, in order to bring interpretation from bench to bedside, it is important that methodology provides discriminatory evidence for individual patients and not just evidence of modest genetic effects between large cohorts.

We describe the implementation of GenePy representing a novel alternative to examine genomic data that provides a quantitative measure of the combined loading of mutation across each gene for each individual. The scoring system has the freedom to harness the intrinsic properties of any user-defined variant-level deleteriousness metric. By summing across genes, GenePy further integrates biological information on frequency and zygosity and when being used to examine between genes or subsets thereof, should be corrected for gene length.

Different measures of deleteriousness impact the coefficient of variation in the GenePy scoring system but as yet none are proven superior. The logarithmic distribution confers weight to rare pathogenic variants and these are additive across a gene and theoretically limited only by the number of variant sites within that gene. GenePy returns a score of zero for the majority of genes for any one individual - this reflects the sparse nature of genomic data and is exacerbated when considering whole exome sequencing data where historical negative selection has limited variation in regions that code for proteins.

We provide proof of principle that testing GenePy scores with a non-parametric statistical test improves sensitivity to detect clinically meaningful gene perturbations. Such performance compares favourably against the most commonly applied gene based association test optimised for small data sets (SKAT-O). Superiority to detect the subtle effects of genes in complex disease is likely attributable to the additional modelling of innate biological features of mutations.

Power to determine significant GenePy score differences between IBD patient and control groups was consistent across sixteen different metrics of variant deleteriousness whereby all concordantly reported a similar level of significance despite differing underlying principles. It is noteworthy that the M-CAP deleteriousness metric that enriches for very deleterious, rare variants proved most significant in our specific test case (although this metric annotated fewer genes than other deleteriousness metrics). This result may suggest a more important role for rare variants in the *NOD2* gene that went largely undetected through GWAS studies. Recent publications have similarly evidenced an important role for rare variants in select patients with IBD [61–64]. While GenePy scores generated using M-CAP metric returned the most significant difference in CD patients compared to controls, it is likely that no metric will prove optimal in all situations. The GenePy scoring system can simply accommodate new and improved variant deleteriousness metrics that are constantly evolving with more widespread use and interpretation of NGS data.

We demonstrated the ability of GenePy to model biological variability from next generation sequencing data on two additional common complex disorders, showing its simple implementation and flexible application to different scenarios. In a Parkinson’s Disease (PD) cohort of very modest sample size compared to contemporary GWAS studies, GenePy successfully identified association with the *PINK1* gene but failed to reach significance for five other known genes when looking across the entire distribution of scores. SKAT-O did not return significant associations with any of the six genes. Interestingly, restricting the analysis to the extreme distribution scores in the case/control comparison framework, GenePy did detect association for four of the six PD genes. This compares well against the SNV association tests within these known genes where only two genes (*LRRK2* and *PINK1*) harboured SNVs that achieved nominal significance without correction for the additional tests incurred by such an approach.

When testing GenePy performances against SKAT-O within the glaucoma cohort, neither SKAT-O or comparison of the entire GenePy distribution between cases and controls could discriminate significant differences between the POAG and non-POAG groups. However, by restricting the analysis to the extreme tail of the distribution, GenePy was able to determine a statistical difference presumably driven by only a minority of patients in whom disease is mediated by the *MYOC* gene.

In addition to identifying genes harbouring statistically significant different mutational loadings between case and control groups, selecting samples from the extreme distribution of GenePy scores concurrently identifies the specific individuals whose disease is (partially) explained by these genes and so facilitates clinical translation.

As with all large-scale data, GenePy scoring is dependent upon data integrity and elimination of systematic bias or technical artefacts. High quality individual DNA samples must be sequenced to sufficient depth to return confident variant calls. For larger scale analyses using multiple samples, parity of capture kits, sequencing platforms and informatic pipelines must be ensured. While these pre-processing quality control steps and generation of the multi-calling VCF file represent the highest computational burden, GenePy score calculation on cleaned vcf files is amenable to batching and computationally trivial.

Many of the currently available deleteriousness scores implemented herein fail to annotate synonymous, splicing or protein truncating variation. While we arbitrarily imposed maximum deleteriousness scores to protein truncating mutations, we standardised the set of variants examined across metrics by excluding synonymous and splicing variants from this analysis. Deleteriousness metrics based on conservation alone are calculable for all genomic variation and could be implemented for the assessment sliding windows of non-coding regions derived from whole genome sequencing. Due to association testing in Caucasian samples only, we restricted allele frequency annotation to that ethnic group. Arguably, there is merit in implementation of global allele frequency estimates or those from more ancestrally diverse populations.

Further refinements of the GenePy scoring system might be realised by integration of gene essentiality [65] (and conversely gene redundancy) or gene damage indices (GDI) [66]. Long read NGS data enabling the discrimination of gametic phase would substantially advantage integration of inheritance models and haploinsufficiency.

## Conclusions

The key advantage of GenePy is its provision of a continuous quantitative measure of biological integrity of a gene within individuals, resulting in a score that is easily integrated into downstream analyses. GenePy scores are not dependent on cohort size and can be calculated and assessed on per-patient patient basis. GenePy scores are suited to pathway analyses where scores can be overlaid and summed across defined molecular cascades. This enables users to assess the combinatorial effect of variants in multiple genes involved in complex diseases. For the particular assessment of complex disease, machine learning tools that integrate multi-omic and extensive biomarker ‘big data’ to determine cryptic patterns are increasingly applied. Currently, all machine learning applications are obliged to incorporate genetic data derived from NGS analyses on a variant-by-variant basis and most do so in either a binary (present/absent) manner or through counting for allelic load (0, 1 or 2) [67]. Both approaches ignore much of the additional biological information already available. Furthermore, these methods often impose arbitrary and subjective filters or thresholds for the inclusion of variants (e.g. frequency) that may be incorrect for Mendelian disease and will certainly reduce power for complex disease. GenePy reduces the dimensionality of genomic data from multiple SNVs within a single gene to the resolution of a single gene. This reduces the number of tests to be performed and impacts statistical power in small cohort studies. GenePy facilitates integration with other ‘omics data that also reports at the level and resolution of a gene e.g. transcriptomic, metabolomic proteomic data and so facilitates integration across these contemporary ‘omic approaches in a machine learning and network analysis frameworks. Furthermore, the assessment of individual gene pathogenicity loadings for individual subjects is simple and intuitive in a clinical setting and allows clustering of independent patients each with cumulatively deleterious burden of mutations in a given gene – even when no specific variants are shared between patients – a situation common for sparse genomic data.

Machine learning approaches aim to define patient subgroups on a molecular genetic basis for the advancement of personalised treatment. Such approaches will directly benefit from the refined scores provided by GenePy for the stratification of different patient subgroups*.* The ability to input biologically rich information and the gene and individual level represents an important step change from the more traditional methods of assessing genetic data at the variant and cohort level.

### Availability and requirements

Project name: GenePy.

Project home page: https://github.com/UoS-HGIG/GenePy

Operating system(s): Unix.

Programming language: Bash, Python 2.7.

Other requirements: GATK 3.x, Annovar.

License: GNU GPL.

Any restrictions to use by non-academics: no licence needed.

## Additional file

Additional file 1: **Table S1.** All single nucleotide variants in the *NOD2* gene used in GenePy validation. **Figure S1.** Median whole gene GenePy_uncorrected_ score profiles observed across the cohort of 508 patients with WES data depicted separately for each of the sixteen deleteriousness metrics. **Figure S2.** Median whole gene GenePy_cgl_ score profiles observed across the cohort of 508 patients with WES data depicted separately for each of the sixteen deleteriousness metrics. **Figure S3.** Ethnicity imputation. **Figure S4.** GenePy scores profiles for the *NOD2* gene in the CD and control groups for each of the sixteen implemented deleteriousness metrics. (DOCX 1054 kb)

## Abbreviations

CD: Crohn’s disease
CGL: Corrected by gene length
CV: Coefficient of variation
GATK: Genome Analysis Toolkit
GWAS: Genome-wide association studies
IBD: Inflammatory bowel disease
IBDU: IBD undetermined
NGS: Next-generation sequencing
PD: Parkinson’s disease
POAG: Primary open angle glaucoma
SKAT-O: Sequence kernel association optimal unified test
SNV: Single nucleotide variant
UC: Ulcerative colitis
VCF: Variant calling format
WES: Whole exome sequencing
