# Supplementary material for: GenePy - a score for estimating gene pathogenicity in individuals using next-generation sequencing data
Source: BMC Bioinformatics. 2019 May 16;20:254. doi: 10.1186/s12859-019-2877-3 (PMC6524327; doi:10.1186/s12859-019-2877-3)
Supplement: Supplementary file 1 — Table S1. All single nucleotide variants in the NOD2 gene used in GenePy validation. Figure S1. Median whole gene GenePyuncorrected score profiles observed across the cohort of 508 patients with WES data depicted separately for each of the sixteen deleteriousness metrics. Figure S2. Median whole gene GenePycgl score profiles observed across the cohort of 508 patients with WES data depicted separately for each of the sixteen deleteriousness metrics. Figure S3. Ethnicity imputation. Figure S4. GenePy scores profiles for the NOD2 gene in the CD and control groups for each of the sixteen implemented deleteriousness metrics. (DOCX 1054 kb) [file 12859_2019_2877_MOESM1_ESM.docx]

**Additional file**

**Additional file 1: Table S1. All single nucleotide variants in the *NOD2* gene used in GenePy validation.** Statistical significance was assessed through a Cochran-Armitage trend test using Plink v1.9 only for common variants (MAF >0.05). Significant associations smaller than 1 x 10^-2^ or smaller than 5 x 10^-2^ are highlighted by two (**) or one (*) asterisks respectively. p-values are not corrected for multiple testing.

| **Chr** | **POS** | **Ref. allele** | **Alt. allele** | **MAF** | **Function†** | **Nucleotide change** | **Amino acid change** | **Controls vs IBD** | **Controls vs UC** | **Controls vs CD** |
| --- | --- | --- | --- | --- | --- | --- | --- | --- | --- | --- |
| chr16 | 50699512 | C | A | 1.18E-03 | NS | c.C17A | p.A6D | . | . | . |
| chr16 | 50699554 | C | T | 2.30E-03 | NS | c.C59T | p.S20L | . | . | . |
| chr16 | 50699710 | C | T | 1.15E-03 | NS | c.C215T | p.A72V | . | . | . |
| chr16 | 50699948 | C | G | 0.356 | SYN | c.C453G | p.S151S | 0.236 | 0.551 | 0.209 |
| chr16 | 50707880 | C | T | 3.66E-03 | NS | c.C485T | p.T162M | . | . | . |
| chr16 | 50710654 | T | G | 2.74E-03 | NS | c.T662G | p.L221R | . | . | . |
| chr16 | 50710713 | C | T | 0.316 | NS | c.C721T | p.P241S | 0.030* | 0.422 | 0.007** |
| chr16 | 50710777 | A | G | 4.70E-03 | NS | c.A785G | p.N262S | . | . | . |
| chr16 | 50710842 | C | T | 1.17E-03 | NS | c.C850T | p.R284W | . | . | . |
| chr16 | 50711028 | C | T | 1.17E-03 | NS | c.C1036T | p.R346C | . | . | . |
| chr16 | 50711101 | C | T | 1.17E-03 | NS | c.C1109T | p.P370L | . | . | . |
| chr16 | 50711203 | C | T | 4.68E-03 | NS | c.C1211T | p.S404L | . | . | . |
| chr16 | 50711204 | G | T | 1.17E-03 | SYN | c.G1212T | p.S404S | . | . | . |
| chr16 | 50711231 | C | T | 1.17E-03 | SYN | c.C1239T | p.T413T | . | . | . |
| chr16 | 50711288 | C | T | 0.316 | SYN | c.C1296T | p.R432R | 0.053 | 0.599 | 0.012* |
| chr16 | 50711492 | C | G | 1.17E-03 | SYN | c.C1500G | p.P500P | . | . | . |
| chr16 | 50711514 | C | T | 1.17E-03 | SYN | c.C1522T | p.L508L | . | . | . |
| chr16 | 50711600 | C | T | 1.17E-03 | SYN | c.C1608T | p.Y536Y | . | . | . |
| chr16 | 50711672 | T | G | 0.355 | SYN | c.T1680G | p.R560R | 0.300 | 0.799 | 0.169 |
| chr16 | 50711699 | G | A | 1.13E-03 | SYN | c.G1707A | p.T569T | . | . | . |
| chr16 | 50711744 | C | T | 0.018 | SYN | c.C1752T | p.A584A | . | . | . |
| chr16 | 50711811 | A | G | 1.13E-03 | NS | c.A1819G | p.R607G | . | . | . |
| chr16 | 50711867 | G | A | 1.13E-03 | SYN | c.G1875A | p.S625S | . | . | . |
| chr16 | 50712015 | C | T | 0.06 | NS | c.C2023T | p.R675W | 0.408 | . | 0.037* |
| chr16 | 50712018 | C | T | 9.05E-03 | NS | c.C2026T | p.R676C | . | . | . |
| chr16 | 50712034 | G | A | 1.13E-03 | NS | c.G2042A | p.R681H | . | . | . |
| chr16 | 50712049 | G | A | 4.53E-03 | NS | c.G2057A | p.R686H | . | . | . |
| chr16 | 50712058 | G | A | 1.13E-03 | NS | c.G2066A | p.R689H | . | . | . |
| chr16 | 50712085 | C | G | 1.13E-03 | NS | c.C2093G | p.A698G | . | . | . |
| chr16 | 50712141 | C | T | 1.13E-03 | NS | c.C2149T | p.R717W | . | . | . |
| chr16 | 50712175 | C | T | 4.53E-03 | NS | c.C2183T | p.A728V | . | . | . |
| chr16 | 50712243 | G | A | 1.13E-03 | NS | c.G2251A | p.E751K | . | . | . |
| chr16 | 50712288 | G | A | 4.53E-03 | NS | c.G2296A | p.V766M | . | . | . |
| chr16 | 50712317 | G | T | 2.26E-03 | SYN | c.G2325T | p.V775V | . | . | . |
| chr16 | 50716594 | G | A | 1.12E-03 | NS | c.G2389A | p.D797N | . | . | . |
| chr16 | 50716899 | A | G | 1.16E-03 | NS | c.A2474G | p.N825S | . | . | . |
| chr16 | 50722629 | G | C | 0.015 | NS | c.G2641C | p.G881R | . | . | . |
| chr16 | 50722660 | C | A | 1.13E-03 | NS | c.C2672A | p.A891D | . | . | . |
| chr16 | 50723365 | G | A | 0.085 | NS | c.G2782A | p.V928I | 0.079 | 0.159 | 0.123 |
| chr16 | 50723375 | A | G | 1.12E-03 | NS | c.A2792G | p.E931G | . | . | . |
| chr16 | 50725494 | A | G | 1.14E-03 | NS | c.A2807G | p.E936G | . | . | . |
| chr16 | 50729867 | G | GC | 0.039 | FSI | c.2936dupC | p.A979fs | . | . | . |

† NS = nonsynonymous SNV, SYN = synonymous SNV, FSI = frameshift insertion

**Additional file 1: Figure S1. Median whole gene GenePy_uncorrected_ score profiles observed across the cohort of 508 patients with WES data depicted separately for each of the sixteen deleteriousness metrics.** For ease of comparison, x-axes are truncated at scores of 10 (maxima are described in Table 2). Bin size was set to 0.01 with the first bin shown 0.0.1-0.02. Grey dashed lines represent the standard deviation of each bin.

**
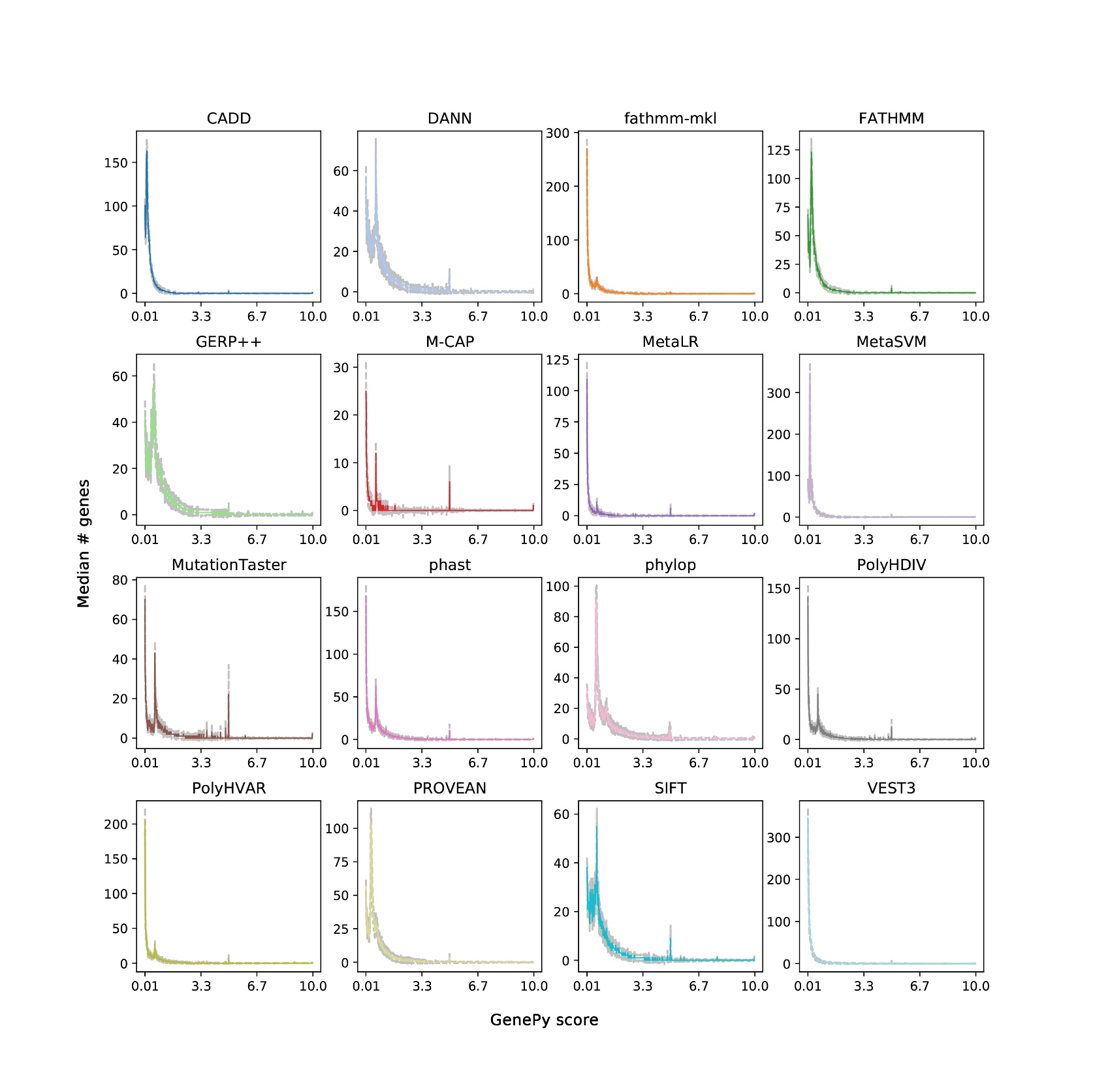
**

**Additional file 1: Figure S2. Median whole gene GenePy_cgl_ score profiles observed across the cohort of 508 patients with WES data depicted separately for each of the sixteen deleteriousness metrics.** For ease of comparison, x-axes are truncated at scores of 10 (maxima are described in Table 2). Bin size was set to 0.01 with the first bin shown 0.0.1-0.02. Grey dashed lines represent the standard deviation of each bin.

**
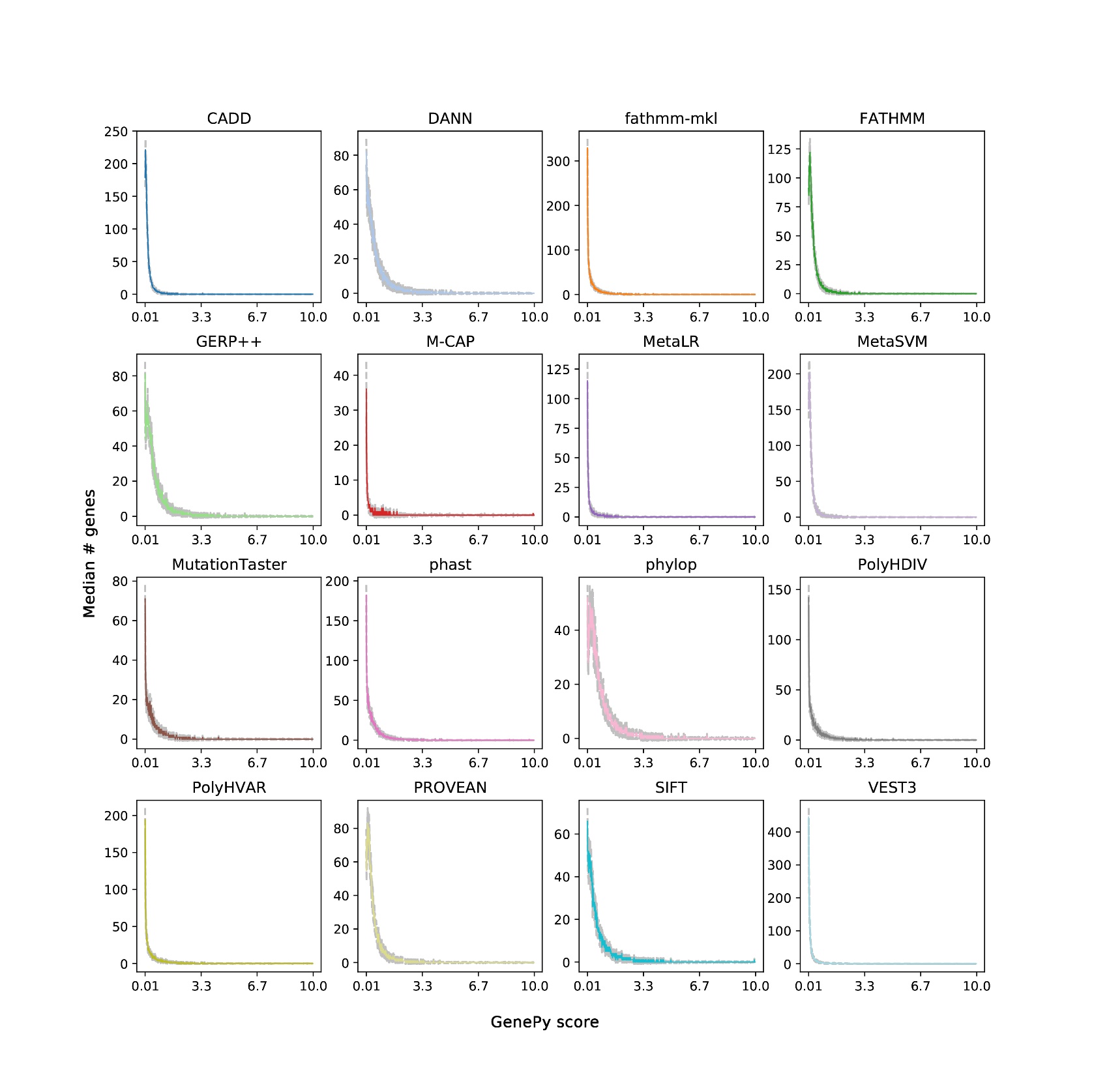
**

**Additional file 1: Figure S3. Ethnicity imputation.** Principal component analysis for the imputation of sample ethnicity. Small dots represent individuals from the 1000 Genomes project used as background. Larger solid dots represent study's individuals coloured according to the imputation result. In order to prevent selection bias, downstream analyses were restricted to Caucasian individuals only. The assessment of the ethnicity was performed modelling 2504 individuals from the 1000 Genomes Project alongside 508 individuals from this study. The multi-sample VCF for our cohort was lifted-over to hg19 reference genome build and then analysed using the Peddy software (<https://github.com/brentp/peddy>) for ethnic imputation. Through Peddy, it was possible to calculate the identity-by-state of all possible sample pairs to impute their ethnicity.


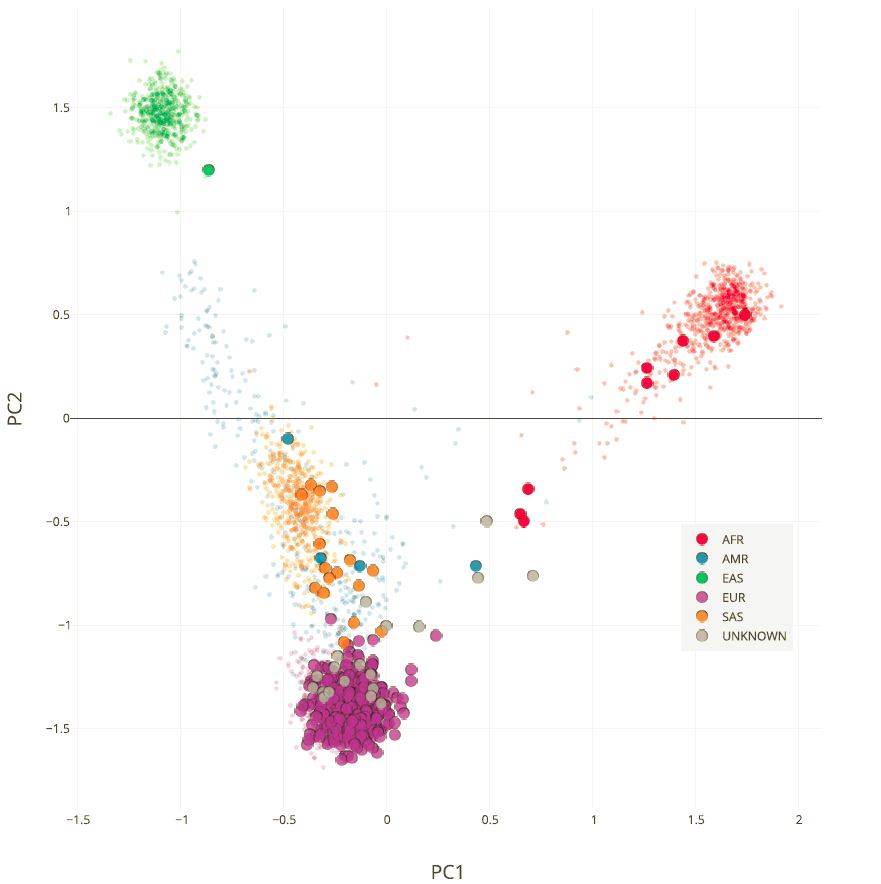


**Additional file 1: Figure S4. GenePy scores profiles for the *NOD2* gene in the CD and control groups for each of the sixteen implemented deleteriousness metrics.** X-axis indicates GenePy scores grouped in bins of size 0.01 with the first bin shown 0.01-0.02. The y-axis shows the observed frequency of GenePy scores across the CD and control cohorts.

**
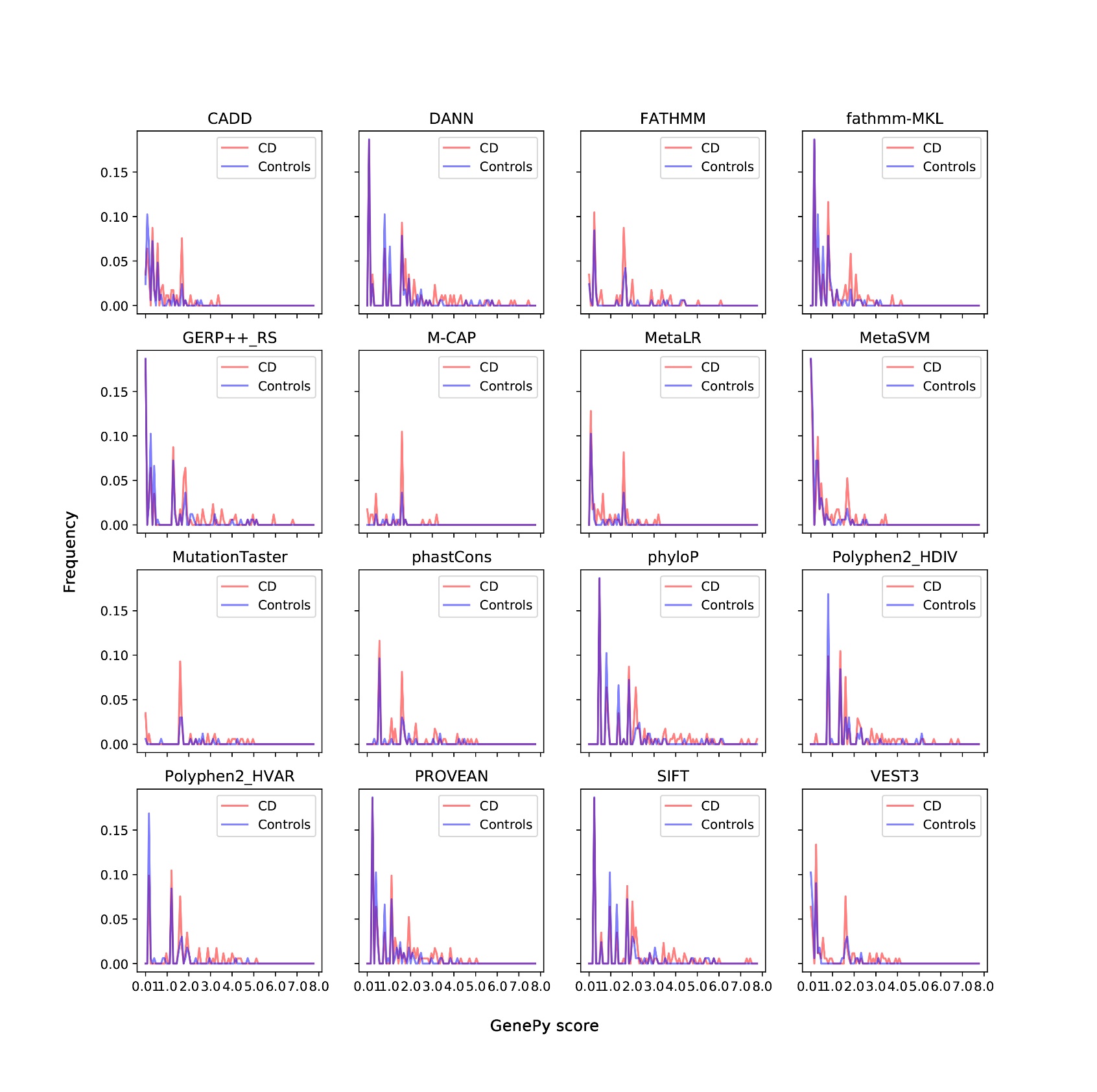
**
